# Supplementary material for: Establishment of Pancreatobiliary Cancer Zebrafish Avatars for Chemotherapy Screening
Source: Cells. 2021 Aug 13;10(8):2077. doi: 10.3390/cells10082077 (PMC8393525; doi:10.3390/cells10082077)
Supplement: Supplementary file 1 [file cells-10-02077-s001.zip › cells-1330769-supplementary.pdf]

Figure S1

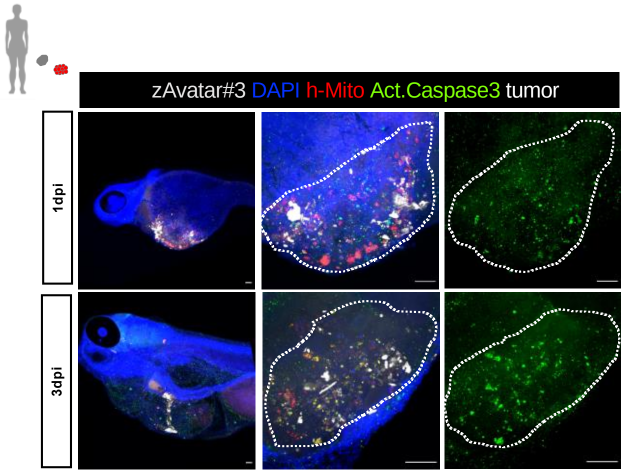

Figure S1 – zAvatars can induce cardiac/abdominal edema. Representative examples of edema in zAvatar#3 fixed at 1dpi and 3dpi, subjected to whole-mount immunofluorescence and imaged by confocal fluorescent microscopy. For all images, tumor cells are labeled in white (Deep Red), activated Caspase3 in green, human-mitochondria in red and DAPI in blue. Scale bars represent 50µm. All images are anterior to the left, posterior to right, dorsal up and ventral down.

Figure S2

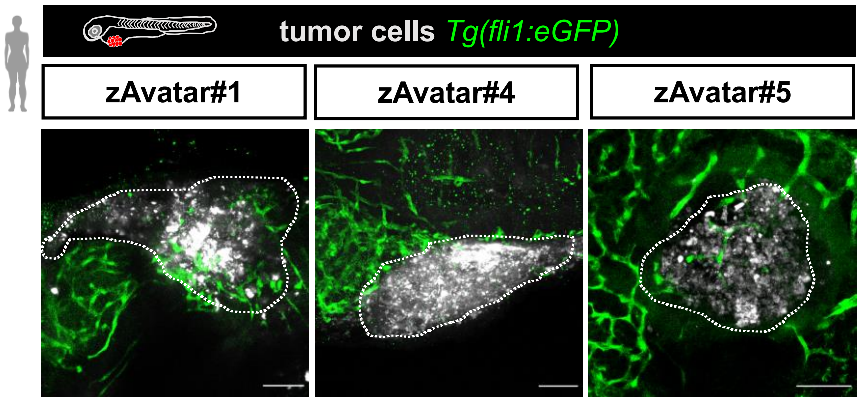

Figure S2. Pancreatic tumors can induce angiogenesis in zebrafish Avatars. Representative examples of endothelial vessel recruitment observed in zAvatar#1, #4 and #5 at 3 dpi, which were subjected to whole-mount immunofluorescence and imaged by confocal fluorescent microscopy. Tumor cells were labeled in white (Deep Red) and injected into zebrafish Tg*(fli1:eGFP)*, whose endothelial vessels are labelled in green.

Figure S3

		Cytopathology									zPDX	
Giemsa Slide ID	Dx extended	cellularity	cohesiveness	naked nuclei	background					other comments	engraftment	angiogenesis
					nuclear tangles	necrosis	stromal fragments	inflammatory cells	apoptotic debris			
#1	PDAC	-	-	-	-	-	-	-	-		70%	yes
#2	PDAC	high	cohesive	20-50%	few	minimal	few	abundant (macrophages)	few	poligonal cells predominant	42%	yes
#3	PDAC	low	poorly cohesive	abundant	abundant	moderate	abundant	abundant (macrophages)	abundant	a lot of cell debris	edema	-
#4	ampullary type	moderate	cohesive	few (<10%)	few	minimal	few	few	few	columnar cells predominant	42%	yes
#5	ampullary type	high	cohesive	20-50%	few	minimal	few	few	few	marked cellular atypia		
#6	distal cholangiocarcinoma	moderate	poorly cohesive	20-50%	moderate	moderate	few	few	moderate	marked cellular atypia; bacteria	edema	-

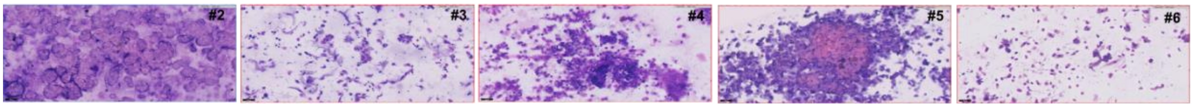

Figure S3. Cytopathologic features of human pancreatobiliary tumors used to establish zAvatars. After dissociation from the original tumor samples, smears were performed and stained with May-Grunwald-Giemsa for quality control analysis. Cellularity, cohesiveness, necrosis and presence of inflammatory were some of the parameters analyzed. We were not able to analyze smears for patient#1.

Table S1. Stock and working concentrations in patients of the antineoplastic drugs used.

Drug	Stock concentration	Working concentration
5-FU	50 mg/mL	426.2 μM
Oxaliplatin	5 mg/mL	8.1 μM
Irinotecan	20 mg/mL	8 μM
Folinic Acid	10 mg/mL	18.5 μM
Gemcitabine	40 mg/mL	160 μM
Paclitaxel	6 mg/mL	475 μg/mL

Table S2. Composition of media used for the generation of zAvatars.

	Reagent	Supplier	Final concentration
**Collection medium**	Advanced DMEM/F-12	Gibco	-
	Penicillin-Streptomycin	Sigma-Aldrich	100U/mL
	Amphotericin B solution	Sigma-Aldrich	100μg/mL
	Kanamycin solution	Sigma-Aldrich	100μg/mL
	Gentamicin solution	Sigma-Aldrich	500μg/mL
	Amoxicillin/Clavulanic acid	Clavepen	220μg/mL
	Metronidazole	Braun	80μg/mL
**Mix 1**	DMEM F12	Gibco	
	HEPES	ThermoFisher	1%
	Glutagro supplement	Corning	1%
	FBS (fetal bovine serum)	Gibco	40%
	Primocin	Invivogen	100μg/mL
	Anoikis inhibitor	Sigma-Aldrich	10μM
	Putrescine	Sigma-Aldrich	10μg/mL
	p38 inhibitor	Sigma-Aldrich	10μM
	Epidermal Growth Factor (EGF)	PeproTech	50ng/mL
	N-acetylcysteine	Sigma-Aldrich	1mM
	Insulin-transferrin-selenium	Corning	1%
	Nicotinamide	Sigma-Aldrich	10mM
**Mix2**	Ethylenediaminetetraacetic acid (EDTA)	Sigma	2mM
	Liberase TM	Roche	50ug/mL
	DNase	ThermoFisher	5U/mL
	Cell tracker Deep Red	Life Technologies	1μL/mL
